# Dephosphorylation and mitochondrial translocation of cofilin sensitizes human leukemia cells to cerulenin-induced apoptosis via the ROCK1/Akt/JNK signaling pathway

**DOI:** 10.18632/oncotarget.7994

**Published:** 2016-03-08

**Authors:** Yanhao Zhang, Ruoqiu Fu, Yanxia Liu, Jing Li, Hongwei Zhang, Xiaoye Hu, Yibiao Chen, Xin Liu, Yunong Li, Ping Li, Ehu Liu, Ning Gao

**Affiliations:** ^1^ College of Pharmacy, 3rd Military Medical University, Chongqing, China; ^2^ State Key Laboratory of Natural Medicines (China Pharmaceutical University), Nanjing, China

**Keywords:** cofilin, ROCK1, Akt, cerulenin, apoptosis

## Abstract

In this study, we determined that cerulenin, a natural product inhibitor of fatty acid synthase, induces mitochondrial injury and apoptosis in human leukemia cells through the mitochondrial translocation of cofilin. Only dephosphorylated cofilin could translocate to mitochondria during cerulenin-induced apoptosis. Disruption of the ROCK1/Akt/JNK signaling pathway plays a critical role in the cerulenin-mediated dephosphorylation and mitochondrial translocation of cofilin and apoptosis. *In vivo* studies demonstrated that cerulenin-mediated inhibition of tumor growth in a mouse xenograft model of leukemia was associated with mitochondrial translocation of cofilin and apoptosis. These data are consistent with a hierarchical model in which induction of apoptosis by cerulenin primarily results from activation of ROCK1, inactivation of Akt, and activation of JNK. This leads to the dephosphorylation and mitochondrial translocation of cofilin and culminates with cytochrome c release, caspase activation, and apoptosis. Our study has revealed a novel role of cofilin in the regulation of mitochondrial injury and apoptosis and suggests that cerulenin is a potential drug for the treatment of leukemia.

## INTRODUCTION

Fatty acid synthase (FAS) is a metabolic enzyme that catalyzes the terminal steps in long chain saturated fatty acid synthesis [1]. The metabolic properties of cancer cells differ from those of normal cells in that cancer cells are more dependent on fatty acid synthesis for proliferation [2]. FAS expression in normal cells is generally very low or undetectable whereas it is highly expressed in most human cancer cells [3]. This difference suggests that targeting metabolic enzymes such as FAS could improve the efficacy of cancer therapy. Recent studies using pharmacologic inhibitors against FAS have shown that inhibition of FAS activity results in severe growth arrest and apoptosis in various types of tumor cells [4]. Several FAS inhibitors including cerulenin, C75, orlistat, C93, and GSK 837149A have shown antitumor activity [5]. Cerulenin is one of the most well-studied inhibitors of FAS and is a natural product of Cephalosporium caerulens [4]. Cerulenin inhibits tumor growth and selectively induces apoptosis in a variety of cancer cell lines through multiple mechanisms including inactivation of PI3K/Akt pathway, activation of JNK, activation of p53, and repression of the *HER2* gene [6–9]. Previous studies have shown that cerulenin induces apoptosis in human leukemia HL60 cells through fatty acid starvation [10]. However, the molecular mechanisms responsible for cerulenin-induced apoptosis in human leukemia cells have not been defined.

Cofilin is a member of the ADF/cofilin family, which regulates actin dynamics by increasing the rate of actin depolymerization and facilitating turnover [11]. Recently, the activities of actin regulatory proteins such as cofilin have been shown to play critical roles in the regulation of apoptosis in mammalian cells [12]. Recent data have suggested that cofilin translocation to the mitochondria is necessary for the opening of the mitochondrial permeability transition pore, and the subsequent release of cytochrome c and initiation of apoptosis [13]. Cofilin activity is inhibited by phosphorylation of the serine residue at position 3 (Ser 3) and induced by dephosphorylation at Ser 3 [14, 15]. Several protein kinases (e.g., LIM-kinases and testicular protein kinases) and phosphatases (e.g., slingshot family protein phosphatases, PP1, and PP2A) are responsible for cofilin phosphorylation and dephosphorylation at Ser 3 [14, 15]. Only dephosphorylated cofilin translocates to the mitochondria after induction of apoptosis [13].

ROCK1 belongs to a family of serine/threonine kinases that are activated by Rho GTPases or caspase-3 cleavage of the C-terminal auto-inhibitory domain away from the kinase active site [16, 17]. Recent studies have shown that ROCK1 regulates apoptosis in various cell types and animal models of diseases [17–19]. Additionally, ROCK1 regulates the dephosphorylation and mitochondrial translocation of cofilin, which is responsible for the induction of apoptosis [18]. However, the precise mechanism by which ROCK1 regulates the phosphorylation state of cofilin during mitochondrial injury and apoptosis is unclear.

In the present study, we found that cerulenin, a natural product inhibitor of FAS, induced mitochondrial injury and apoptosis in human leukemia cells through the mitochondrial translocation of cofilin. Only dephosphorylated cofilin translocated to mitochondria during cerulenin-induced apoptosis. Mechanistic studies revealed that interruption of the ROCK1/Akt/JNK signaling pathway plays a critical role in the cerulenin-mediated dephosphorylation and mitochondrial translocation of cofilin, and induction of apoptosis. *In vivo* indicated that cerulenin-mediated inhibition of tumor growth in a mouse xenograft model of leukemia was associated with the mitochondrial translocation of cofilin and apoptosis. Our study has revealed a novel role of cofilin in the regulation of mitochondrial injury and apoptosis, and suggests that cerulenin is a potential therapeutic for leukemia.

## RESULTS

### Cerulenin selectively induces mitochondrial injury and apoptosis in human leukemia cells

**Figure 1:**
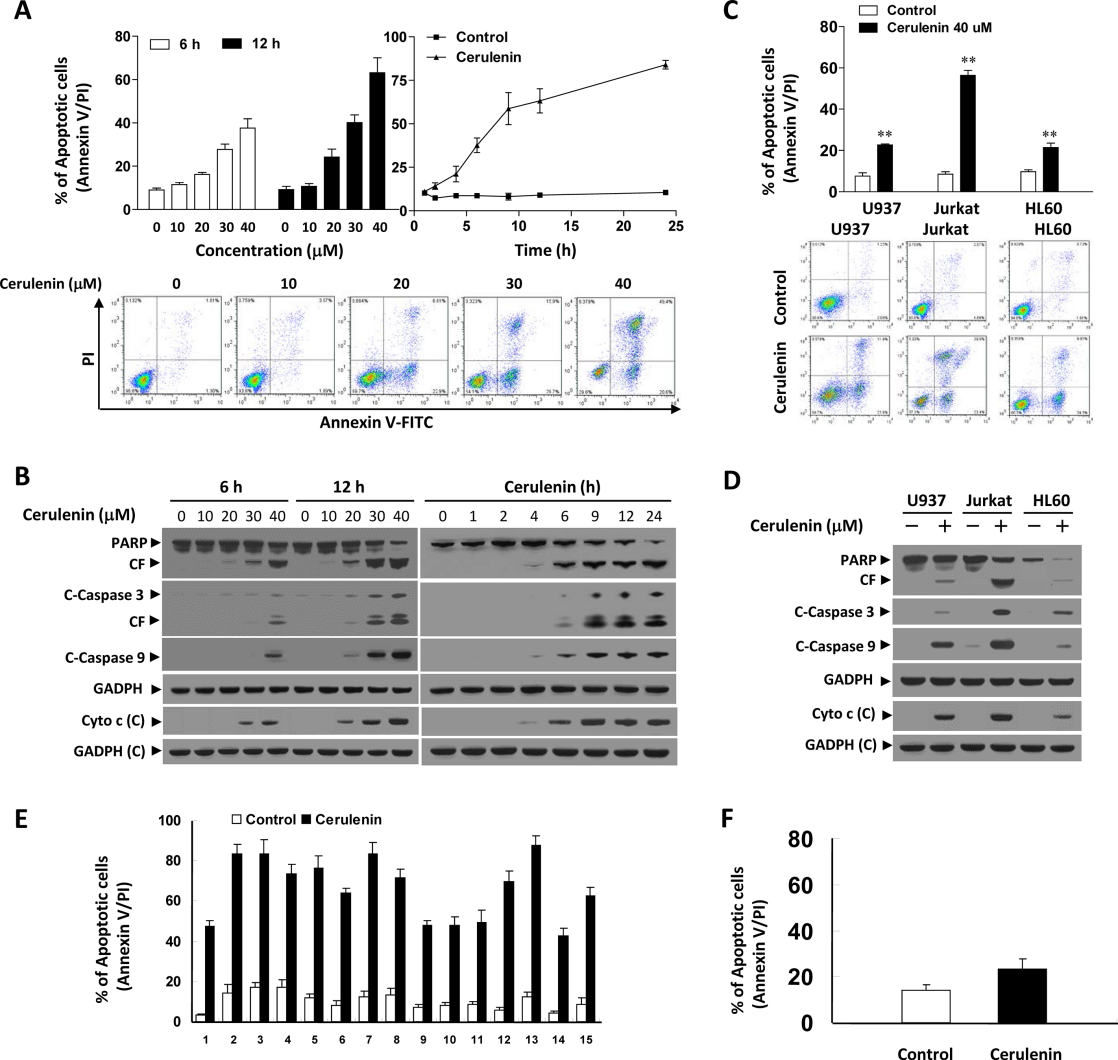
Cerulenin induces apoptosis and caspase activation in human leukemia cells Jurkat cells were treated with various concentrations of cerulenin as indicated for 6 and 12 h, or with 40 μM cerulenin for 1, 2, 4, 6, 9, 12, and 24 h. (**A**) Cells were washed twice with cold PBS and then stained with annexin V/PI. Apoptosis was evaluated using flow cytometry as described in the Materials and Methods. The values obtained from the annexin V/PI assays represent the mean ± s.d. for three separate experiments. Lower panel: Representative fluorescence activated cell sorting (FACS) images. (**B**) After treatment of the cells with the indicated concentrations of cerulenin or incubation for the various times, WCLs and cytosolic fractions (cytochrome c) were prepared and subjected to western blot analysis using antibodies against PARP, cleaved-caspase 3 (C-Caspase 3), C-Caspase 9, and cytochrome c (Cyto c). CF, cleavage fragment; C, cytosolic fractions. U937, Jurkat, and HL-60 cells were treated without or with 40 μM cerulenin for 12 h. (**C**) Cells were stained with annexin V/PI, and apoptosis was evaluated using flow cytometry as described in the Materials and Methods. The values obtained from the annexin V/PI assays represent the mean ± s.d. for three separate experiments. **The values for cells treated with cerulenin were significantly higher than those of control cells based on Student's *t*-tests; *P* < 0.01. Lower panel: Representative FACS images. (**D**) After treatment with cerulenin, WCLs and cytosolic fractions were prepared and subjected to Western blot analysis using antibodies against PARP, C-Caspase 3, C-Caspase 9, and Cyto c. (**E**) Primary leukemia blasts from 15 AML patients were treated with 40 μM cerulenin for 24 h and apoptosis analyzed by flow cytometry analysis using annexin V/PI staining. (**F**) Normal CD34^+^ cells were isolated from a normal bone marrow sample and exposed to 40 μM cerulenin for 24 h. The percentage of apoptotic cells was determined by flow cytometry using annexin V/PI staining. In the western blot analyses, each lane was loaded with 30 μg protein. The blots were subsequently stripped and reprobed with an antibody against GADPH to ensure equivalent loading and transfer. Two additional studies yielded equivalent results.

Dose-response and time course analyses of cerulenin-mediated apoptosis in Jurkat cells are shown in Figure 1A. A modest degree of apoptosis was observed at a cerulenin concentration of 20 μM (6 and 12 h), which increased substantially at concentrations ≥ 30 μM. Exposure of cells to 40 μM cerulenin for varying amounts of time resulted in a moderate increase in apoptosis as early as 6 h after drug exposure. Longer exposure times resulted in increase cell death (59%, 63%, and 84% at 9 h, 12 h, and 24 h, respectively). Western blot analysis revealed that cerulenin induced cleavage/activation of caspases-3 and -9, and degradation of poly ADP ribose polymerase (PARP) in a dose- and time-dependent manner. These events were accompanied by the release of cytochrome c into the cytosol (Figure 1B).

To determine whether the cerulenin-mediated induction of apoptosis was restricted to myeloid leukemia cells, parallel studies were performed in U937 and HL-60 leukemia cells. The apoptotic effects of cerulenin on these cells were similar to those in Jurkat cells (Figure 1C). Additionally, U937 and HL-60 cells exhibited comparable degrees of caspase-3 and -9 cleavage/activation, PARP degradation, and cytochrome c release in response to cerulenin treatment (Figure 1D).

To determine whether cerulenin could also trigger apoptosis in primary human leukemia cells, we isolated these cells from 15 patients with acute myelogenous leukemia (AML) and treated them with 40 μM cerulenin for 24 h. Apoptosis was then analyzed based on annexin V/propidium iodide (PI) staining. Exposure of AML blasts to cerulenin resulted in a significant increase in apoptosis compared to untreated control cells (Figure 1E). In contrast, cerulenin exerted little toxicity toward normal CD34^+^ bone marrow cells (Figure 1F). These results suggested that cerulenin selectively induced mitochondrial injury and apoptosis in transformed and primary human leukemia cells but not in normal hematopoietic cells.

### Cerulenin induces the dephosphorylation and mitochondrial translocation of cofilin

**Figure 2:**
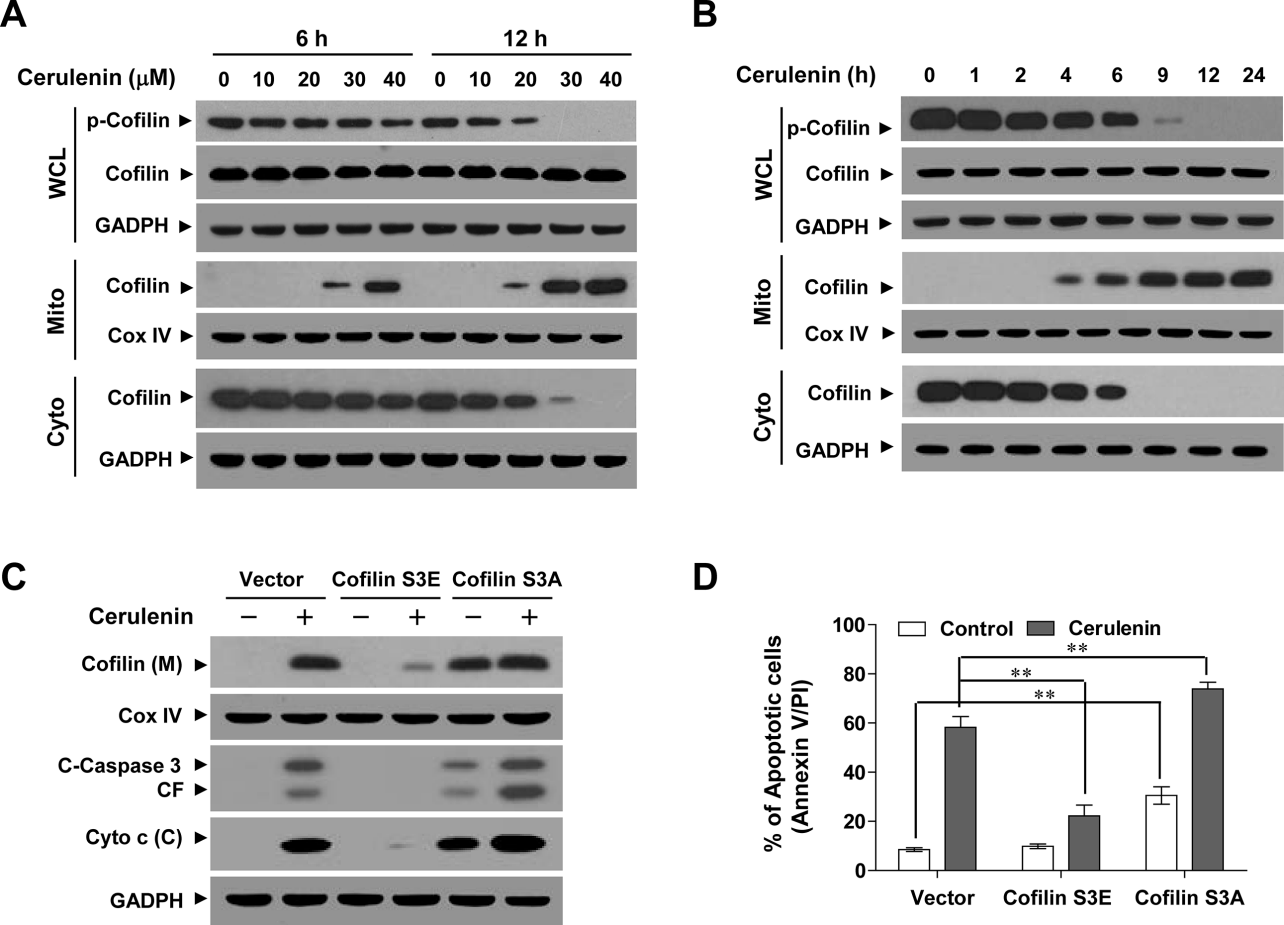
Cerulenin induces the dephosphorylation and mitochondrial translocation of cofilin (**A** and **B**) Jurkat cells were either treated with various concentrations of cerulenin as indicated for 6 and 12 h, or with 40 μM cerulenin for 1, 2, 4, 6, 9, 12, and 24 h. After treatment with cerulenin, WCLs, and both cytosolic (Cyto) and mitochondrial (Mito) fractions were prepared and subjected to western blot analysis using antibodies against p-cofilin and cofilin. Jurkat cells were transfected with control empty vector, human dephosphorylated cofilin (active, S3A), or pseudophosphorylated (inactive, S3E) cofilin for 48 h. (**C**) After treatment with 40 μM cerulenin for 12 h, WCLs, mitochondrial (M), and cytosolic (C) fractions were prepared and subjected to western blot analysis using antibodies against cofilin, C-Caspase 3, and Cyto c. (**D**) The percentage of apoptotic cells was analyzed by flow cytometry using annexin V/PI staining. For western blot analysis, blots were subsequently stripped and reprobed with an antibody against GADPH and Cox IV (mitochondrial fraction) to ensure equivalent loading. Two additional studies yielded equivalent results.

Since mitochondrial translocation of cofilin is an early step in apoptosis [13], we investigated whether mitochondrial translocation of cofilin was necessary for cerulenin to induce apoptosis. Treatment of cells with cerulenin significantly increased the levels of cofilin in mitochondria and decreased the levels of cofilin in the cytosol in a dose- and time-dependent manner (Figure 2A and 2B). Recent studies have indicated that only dephosphorylated cofilin is translocated to mitochondria upon initiation of apoptosis [13]. Therefore, we next investigated whether cerulenin altered the phosphorylation status of cofilin. Treatment of cells with cerulenin resulted in a dose- and time-dependent decrease in the level of phosphorylated cofilin (Ser 3) in whole cell lysates (WCLs) (Figure 2A and 2B).

To determine whether the phosphorylation status of cofilin influenced its ability to translocate to mitochondria and induce injury and apoptosis, two cofilin mutants that mimicked the dephosphorylated and phosphorylated forms were generated by mutagenesis of Ser 3 to either alanine (active; S3A) or glutamic acid (inactive; S3E) as described previously [20]. Overexpression of cofilin S3A enhanced the mitochondrial translocation of cofilin mediated by cerulenin whereas cofilin S3E abolished translocation (Figure 2C). Furthermore, cofilin S3A significantly enhanced, while cofilin S3E reduced, cerulenin-mediated cytochrome c release, caspase 3 activation, and apoptosis (Figure 2C and 2D). Thus, our data indicated that cerulenin-mediated dephosphorylation of cofilin (Ser 3) is required for cofilin translocation to mitochondria and apoptosis.

### Cerulenin induces JNK activation, Akt inactivation, and ROCK1 activation

**Figure 3:**
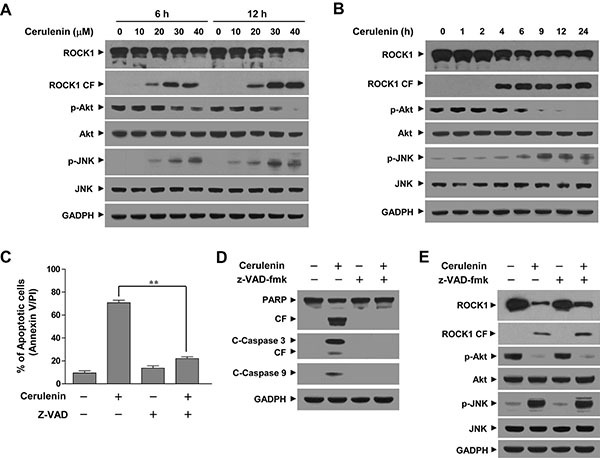
Cerulenin induces ROCK1 activation, Akt inactivation, and JNK activation (**A** and **B**) Jurkat cells were treated with either various concentrations of cerulenin as indicated for 6 and 12 h or with 40 μM cerulenin for 1, 2, 4, 6, 9, 12, and 24 h. After treatment with cerulenin, WCLs were prepared and subjected to western blot analysis using antibodies against ROCK1, phosphorylated Akt (p-Akt), Akt, phosphorylated JNK (p-JNK), and JNK. Jurkat cells were pretreated with the caspase inhibitor z-VAD-fmk (10 μM) for 1 h, and then treated with 40 μM of cerulenin for 12 h. (**C**) Cells were stained with annexin V/PI, and apoptosis was evaluated using flow cytometry as described in the Materials and Methods. The values obtained from the annexin V/PI assays represent the mean ± s.d. for three separate experiments. **The values for cells treated with cerulenin and z-VAD-fmk were significantly lower that those of cells treated with cerulenin alone based on Student's *t*-tests; *P* < 0.01. (**D** and **E**) After treatment with cerulenin in the presence or absence of z-VAD-fmk, WCLs were prepared and subjected to western blot analysis using antibodies against PARP, C-Caspase 3, C-Caspase 9, ROCK1, p-Akt, Akt, p-JNK, and JNK. For western blot analyses, each lane was loaded with 30 μg of protein. The blots were subsequently stripped and reprobed with an antibody against GADPH to ensure equivalent loading. Two additional studies yielded equivalent results.

The effects of cerulenin in Jurkat cells were examined in relation to changes in various signal transduction pathways implicated in the regulation of apoptosis. Exposure of Jurkat cells to cerulenin resulted in decreased ROCK1 levels and increased ROCK1 cleavage in a dose- and time-dependent manner (Figure 3A and 3B). Treatment of cells with cerulenin also resulted in a decrease in phosphorylated Akt (p-Akt) levels and an increase in phosphorylated JNK (p-JNK) levels in a dose- and time-dependent manner (Figure 3A and 3B). In contrast, cerulenin had little or no effect on the expression of total or phosphorylated mTOR, ERK, or p38 MAPK (data not shown). These findings suggested that activation of ROCK1, inactivation of Akt, and activation of the JNK pathway were important for cerulenin-induced mitochondrial injury and apoptosis in leukemia cells.

Because perturbations of ROCK1 activation and Akt inactivation in cerulenin-treated cells could have represented secondary events that stemmed from caspase activation, parallel studies were performed in cells exposed to the broad caspase inhibitor z-VAD-fmk. As anticipated, the addition of z-VAD-fmk blocked cerulenin-induced apoptosis (Figure 3C), cleavage/activation of caspase-3, and -9, and PARP degradation (Figure 3D). However, it did not prevent cerulenin-mediated ROCK1 activation, Akt inactivation, and JNK activation (Figure 3E). These data indicated that cerulenin-mediated perturbations in ROCK1/Akt/JNK signaling and apoptotic regulatory events proceeded through a caspase-independent pathway.

### JNK activation plays a critical role in the cerulenin-mediated dephosphorylation and mitochondrial translocation of cofilin and apoptosis in Jurkat cells

**Figure 4:**
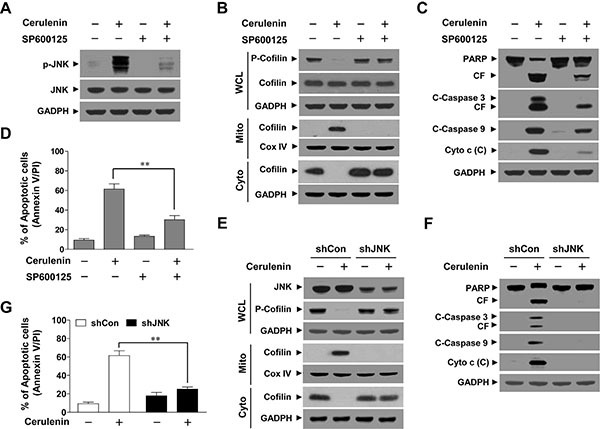
Inhibition of JNK by SP600125 or JNK siRNA abrogated cerulenin-induced apoptosis Jurkat cells were pretreated with 10 μM of the JNK inhibitor SP600125 for 1 h, and then treated with 40 μM of cerulenin for 12 h. (**A**) WCLs were prepared and subjected to western blot analysis using antibodies against p-JNK and JNK. (**B**) WCLs, mitochondrial (Mito), and cytosolic (Cyto) fractions were prepared and subjected to western blot analysis using antibodies against p-cofilin and cofilin. (**C**) WCLs and cytosolic (C) fractions were prepared and subjected to immunoblotting with antibodies against PARP, C-Caspase-3, C-Caspase 9, and cytochrome c (Cyto c). (**D**) Cells were stained with annexin V/PI, and apoptosis was analyzed using flow cytometry as described in the Materials and Methods. The values obtained from the annexin V/PI assays represent the mean ± s.d. for three separate experiments. **The values for cells treated with cerulenin and SP600125 were significantly lower than those obtained for cells treated with cerulenin alone by Student's *t*-test; *P* < 0.01. Jurkat cells were transfected with JNK siRNA oligonucleotides or controls siRNA and incubated for 24 h at 37°C, after which cells were treated with 40 μM of cerulenin for 12 h. (**E**) WCLs, Mito, and Cyto fractions were prepared and subjected to western blot analysis using antibodies against JNK, p-Cofilin, and cofilin. (**F**) WCLs and cytosolic (C) fractions were prepared and subjected to Western blot analysis using antibodies against PARP, C-Caspase-3, and cytochrome c (Cyto c). (**G**) Apoptosis was evaluated using the annexin V/PI assay as described in the Materials and Methods. The values obtained from annexin V/PI assays represent the mean ± s.d. for three separate experiments. **The values for cells treated with cerulenin after transfection with JNK siRNA (shJNK) oligonucleotides were significantly decreased compared to those of control siRNA (shCon)-transfected cells treated with cerulenin based on Student's *t*-tests (*P* < 0.01).

To determine whether JNK activation was important for cerulenin-mediated apoptosis, both a pharmacologic and genetic approach was employed. Co-administration of the JNK inhibitor SP600125 abrogated cerulenin-mediated activation of JNK, based on the diminished p-JNK (Figure 4A). Co-administration of SP600125 also blocked the cerulenin-mediated dephosphorylation and mitochondrial translocation of cofilin (Figure 4B), and attenuated cerulenin-induced cytochrome c release, cleavage/activation of caspase-3 and -9, degradation of PARP, and apoptosis (*P* < 0.01 between combined treatment and cerulenin alone, Figure 4C and 4D). Since SP600125 is not completely specific for JNK, a genetic approach utilizing JNK siRNA was employed [21]. Transient transfection of Jurkat cells with JNK siRNA reduced the expression of JNK to one-third that of control cells (Figure 4E). Knockdown of JNK also abrogated cerulenin-mediated dephosphorylation and mitochondrial translocation of cofilin (Figure 4E). Furthermore, knockdown of JNK significantly attenuated cerulenin-mediated cytochrome c release, cleavage/activation of caspase-3 and -9, degradation of PARP, and apoptosis (Figure 4F and 4G). Collectively, these findings indicated that JNK activation was functionally important for the cerulenin-mediated dephosphorylation and mitochondrial translocation of cofilin, mitochondrial injury, and apoptosis.

### Akt inactivation functions in cerulenin-mediated JNK activation, cofilin dephosphorylation, mitochondrial translocation, and apoptosis in Jurkat cells

**Figure 5:**
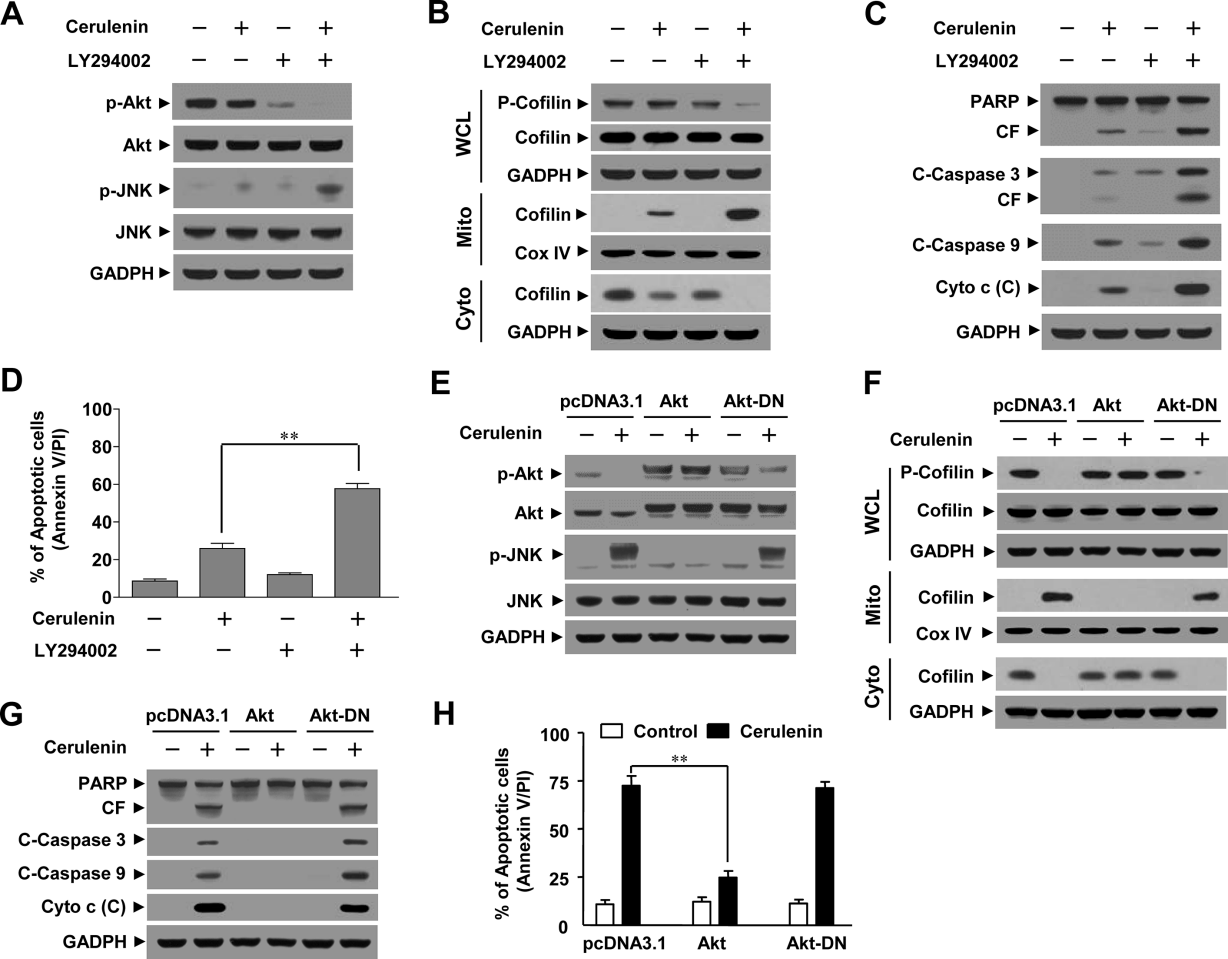
Inhibition of Akt by LY294002 enhanced cerulenin-induced apoptosis while constitutive activation of Akt protected cells from cerulenin-induced apoptosis Jurkat cells were pretreated with 20 μM of LY294002 for 1 h and then treated with 20 μM of cerulenin for 12 h. (**A**) WCLs were prepared and subjected to western blot analysis using antibodies against p-Akt, Akt, p-JNK, and JNK. (**B**) WCLs, mitochondrial (M) and cytosolic (**C**) fractions were prepared and subjected to Western blot analysis using antibodies against p-cofilin, and cofilin. (C) WCLs or cytosolic (C) fractions were also prepared and subjected to western blot analysis using antibodies against PARP, C-Caspase 3, C-Caspase 9, and Cyto c. (**D**) Cells were stained with annexin V/PI and apoptosis was analyzed using flow cytometry as described in the Materials and Methods. The values obtained from annexin V/PI assays represent the mean ± s.d. for three separate experiments. **The values for cells treated with both cerulenin and LY294002 were significantly higher than those of cells treated with cerulenin alone based on Student's *t*-tests (*P* < 0.01). Jurkat cells were stably transfected with empty vector (pcDNA3.1), constitutively active Akt, or dominant negative Akt (Akt-DN) as described in the Materials and Methods. These cells were treated without or with 40 μM of cerulenin for 12 h. (**E**–**G**) WCLs, mitochondrial (M), and cytosolic (C) fractions were prepared and subjected to Western blot analysis using antibodies against p-Akt, Akt, p-JNK, JNK, p-cofilin, cofilin, PARP, C-Caspase-3, C-Caspase-9, and Cyto c. (**H**) Apoptosis was analyzed using annexin V/PI assays as described in the Materials and Methods. The values obtained from the annexin V/PI assays represent the mean ± s.d. for three separate experiments. **The values for Akt cells treated with cerulenin were significantly lower that those of pcDNA3.1 or Akt-DN-transfected cells based on Student's *t*-tests (*P* < 0.01).

Our data implied that inactivation of Akt could play an important role in cerulenin-mediated lethality. To test this possibility, we used the PI3K inhibitor LY294002. Western blot analysis indicated that co-administration of a non-toxic concentration of LY294002 (i.e., 20 μM) and a sub-lethal concentration of cerulenin resulted abrogated Akt phosphorylation and increased JNK activation (Figure 5A). Co-administration of LY294002 significantly enhanced the cerulenin-mediated dephosphorylation and mitochondrial translocation of cofilin (Figure 5B). In addition, co-administration of LY294002 markedly enhanced cerulenin-mediated cytochrome c release, cleavage/activation of caspase-3 and -9, degradation of PARP, and apoptosis (Figure 5C and 5D).

To further assess the functional significance of Akt inactivation in cerulenin-mediated lethality, a constitutively active form of Akt or dominant negative Akt (Akt-DN) were ectopically expressed in Jurkat cells. Western blot analysis revealed marked constitutive myristoylated Akt accompanied by a striking increase in Akt phosphorylation. In contrast to the controls, neither the expression nor phosphorylation of Akt decreased in response to cerulenin treatment (Figure 5E). Interestingly, cerulenin-mediated JNK activation was abrogated in Akt cells, suggesting that Akt inactivation was upstream of JNK activation (Figure 5E). Constitutive activation of Akt blocked the cerulenin-mediated dephosphorylation and mitochondrial translocation of cofilin (Figure 5F). Furthermore, it attenuated cerulenin-mediated cytochrome c release, cleavage/activation of caspase-3 and -9, degradation of PARP, and apoptosis (Figure 5G and 5H). These data indicated that inactivation of Akt was important for the cerulenin-mediated activation of JNK, dephosphorylation and mitochondrial translocation of cofilin, and apoptosis.

### ROCK1 activation plays a critical role in cerulenin-mediated Akt inactivation, JNK activation, cofilin dephosphorylation and mitochondrial translocation, and apoptosis in jurkat cells

**Figure 6:**
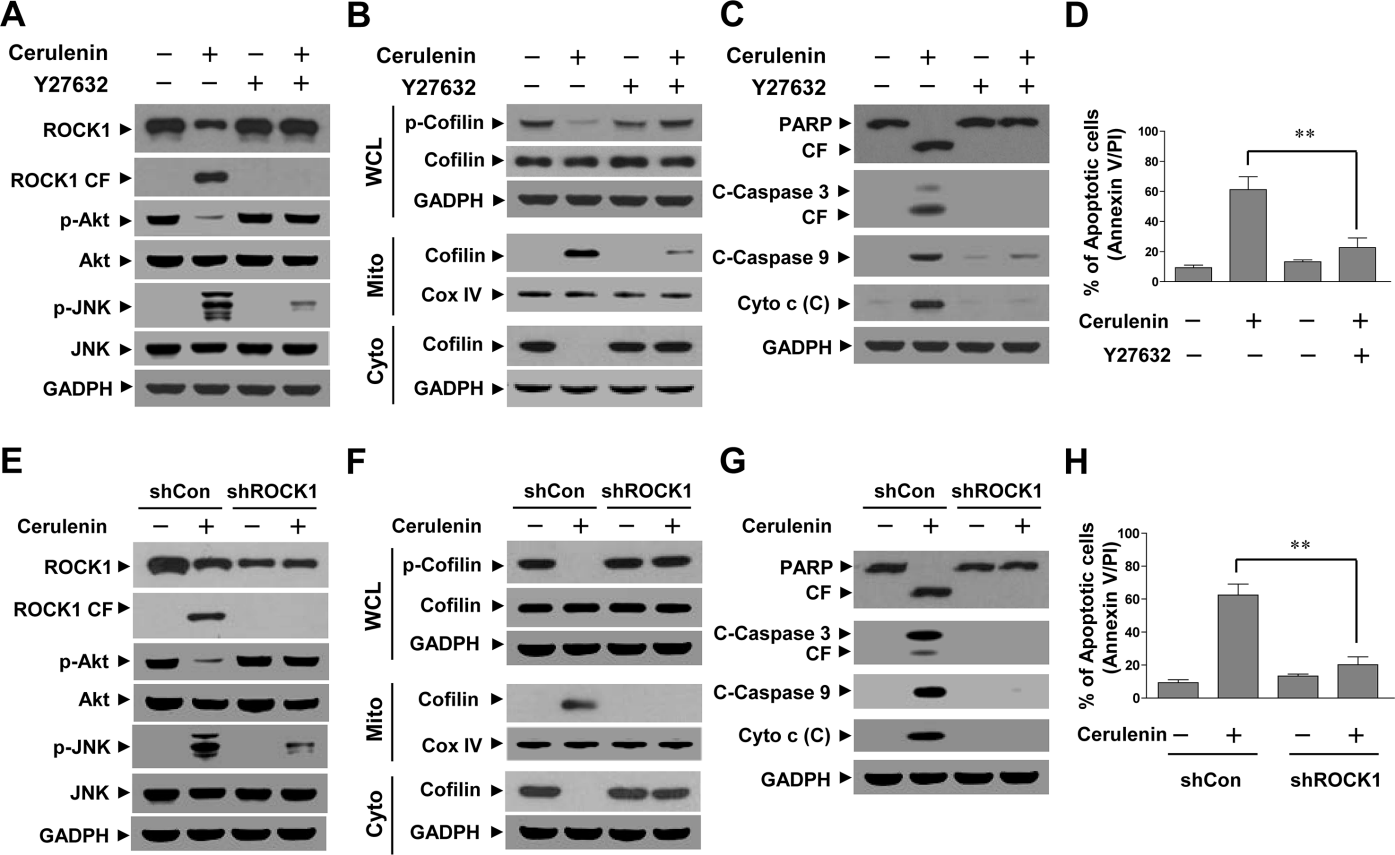
Pharmacological inhibition of ROCK1 or knockdown of ROCK1 with siRNA significantly abrogated cerulenin-induced apoptosis. Jurkat cells were pretreated with 20 μM Y27632 for 2 h and then treated with 40 μM cerulenin for 12 h. (**A**–**C**) WCLs, mitochondrial (Mito), and cytosolic (Cyto) fractions were prepared and subjected to western blot analysis using antibodies against ROCK1, p-Akt, Akt, p-JNK, JNK, p-cofilin, cofilin, PARP, C-Caspase 3, C-Caspase 9, and Cyto c. (**D**) Cells were stained with annexin V/PI, and apoptosis was analyzed using flow cytometry as described in the Materials and Methods. The values obtained from annexin V/PI assays represent the mean ± s.d. for three separate experiments. **The values for cells treated with cerulenin and Y27632 were significantly lower than those of cells treated with cerulenin alone based on Student's *t*-tests (*P* < 0.01). Jurkat cells were infected with lentivirus-containing constructs that encoded scrambled control shRNA (shCon) or human ROCK1-specific shRNA (shROCK1). Stable cell lines were treated without or with 40 μM cerulenin for 12 h. (**E**–**G**) Following treatment, WCLs, Mito, and Cyto fractions were prepared and subjected to western blot assays using the indicated antibodies. (**H**) Apoptosis was evaluated using annexin V/PI assays as described in the Materials and Methods. The values obtained from the annexin V/PI assays represent the mean ± s.d. for three separate experiments. **The values for cells treated with cerulenin after infection with ROCK1 shRNA were significantly lower than those of shCon cells treated with cerulenin based on Student's *t*-tests (*P* < 0.01).

To determine whether ROCK1 activation was critical for cerulenin-mediated apoptosis, we employed the ROCK1 inhibitor Y27632. Western blot analysis indicated that pretreatment of Jurkat cells with Y27632 abrogated cerulenin-induced ROCK1 activation, Akt inactivation, and JNK activation (Figure 6A). Pretreatment of Jurkat cells with Y27632 attenuated cerulenin-mediated cofilin dephosphorylation and mitochondrial translocation (Figure 6B). Consistent with these findings, pretreatment of Jurkat cells with Y27632 also attenuated cerulenin-mediated cytochrome c release, caspase-3 and -9 cleavage/activation, PARP degradation, and apoptosis (Figure 6C and 6D).

We next knocked down ROCK1 expression in Jurkat cells using a ROCK1-specific siRNA. Western blots showed that Jurkat cells transfected with ROCK1 siRNA had reduced expression of total ROCK1 and cleaved ROCK1. Knockdown of ROCK1 also attenuated cerulenin-mediated Akt inactivation and JNK activation (Figure 6E). Knockdown of ROCK1 significantly abrogated cerulenin-mediated cofilin dephosphorylation and mitochondrial translocation (Figure 6F). In addition, knockdown of ROCK1 dramatically abrogated cerulenin-mediated cytochrome c release, caspase-3 and -9 cleavage/activation, PARP degradation, and apoptosis (Figure 6G). These results suggested that the ROCK1/Akt/JNK signaling pathway was functionally important for cerulenin-mediated cofilin dephosphorylation and mitochondrial translocation as well as apoptosis in human leukemia cells.

### Cerulenin inhibits tumor growth and induces apoptosis in a jurkat xenograft mouse model

**Figure 7:**
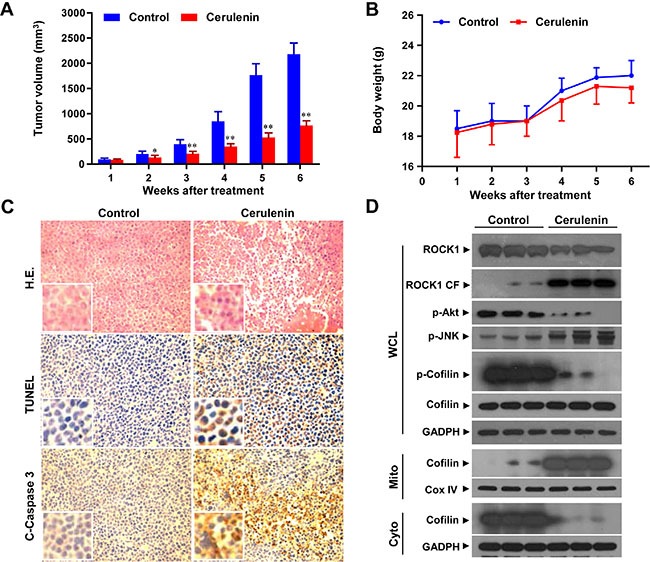
Cerulenin inhibits tumor growth in a jurkat xenograft mouse model Thirty nude mice were inoculated with Jurkat cells (2 × 10^6^ cells/mouse, subcutaneously) and randomly divided into two groups (15 mice/group) for treatment with cerulenin (50 mg/kg, intraperitoneally, daily, five times per week) or with vehicle control as described in the Materials and Methods. (**A**) Tumor volume was assessed as described in the Materials and Methods. Data represent the mean ± s.d. *P < 0.05 or **P < 0.01 compared to the vehicle control. (**B**) Body weight changes of mice treated with cerulenin for 6 weeks. Statistical analysis of the changes in body weight revealed no significant differences between the cerulenin- and vehicle-treated groups. (**C**) Following treatment with cerulenin (50 mg/kg), tumor tissues were sectioned and subjected to H & E staining, TUNEL assays, and immunohistochemistry in order to evaluate cell morphology, apoptosis, and C-Caspase 3 expression. (**D**) Tumors from 3 vehicle control mice and 3 mice treated with cerulenin (50 mg/kg) were harvested and homogenized. WCLs, mitochondrial (Mito) and cytosolic (Cyto) fractions were collected and subjected to western blot analysis using antibodies against ROCK1, p-Akt, p-JNK, p-cofilin, cofilin, Cox IV, and GADPH.

The ability of cerulenin to kill both transformed and primary human leukemia cells *in vitro* led us to evaluate the anti-leukemic activity *in vivo*. Nude mice were inoculated subcutaneously with Jurkat cells and then injected with either vehicle or cerulenin (50 mg/kg, intraperitoneally) for 6 weeks starting 3 days after injection of the leukemia cells. Treatment of mice with cerulenin resulted in modest but significant suppression of tumor growth 2 weeks after drug exposure (*P* < 0.05 vs. vehicle control). These effects became more apparent after 3 and 4 weeks, and were extensive 5 and 6 weeks after drug exposure (*P* < 0.01 vs. vehicle control) (Figure 7A). However, no significant changes in weight (Figure 7B) or other signs of toxicity such as agitation, impaired movement and posture, indigestion or diarrhea, and areas of redness or swelling were observed during the treatment with cerulenin.

To evaluate the effect of cerulenin on morphological changes and induction of apoptosis in tumor sections from Jurkat xenografts, hematoxylin and eosin (H & E) staining, TUNEL, and immunohistochemistry analyses were performed. The sections of Jurkat xenografts from mice treated with cerulenin had fewer cancer cells along with signs of necrosis and infiltration of inflammatory cells (e.g., phagocytic cells) and apoptotic regions (Figure 7C, top panels). Treatment of mice with cerulenin also resulted in a striking induction of tumor cell apoptosis in the tumor cells (i.e., numerous dark brown colored apoptotic cells, Figure 7C, middle panels). Finally, treatment with cerulenin caused a rapid increase in immunoreactivity for cleaved caspase-3, which was also indicative of apoptosis (Figure 7C, bottom panels). These data suggested that cerulenin significantly inhibited tumor growth in a Jurkat xenograft mouse model through induction of apoptosis.

To determine whether the activation of ROCK1 and dephosphorylation and mitochondrial translocation of cofilin was involved in cerulenin-induced apoptosis *in vivo*, we performed western blot analyses. Treatment of mice with cerulenin resulted in decreased ROCK1 levels and increased ROCK1 cleavage (Figure 7D). Treatment with cerulenin also decreased the levels of phosphorylated cofilin in WCLs (Figure 7D). In addition, it resulted in decreased cofilin levels in the cytosolic fraction and increased levels in the mitochondrial fraction (Figure 7D). Such findings suggest that activation of ROCK1 and dephosphorylation and mitochondrial translocation of cofilin could contribute to cerulenin-mediated apoptosis and anti-leukemic effects *in vivo*.

## DISCUSSION

In the present study, we demonstrated that the FAS inhibitor cerulenin potently induces mitochondrial injury and apoptosis in multiple types of human leukemia cell lines and primary human AML cells. This phenomenon was primarily due to the dephosphorylation and mitochondrial translocation of cofilin. Cofilin plays a crucial role in the formation of actin filaments by regulating polymerization and depolymerization [22]. Recent evidence has indicated that the mitochondrial translocation of cofilin is an early step in apoptosis [13]. The translocation of cofilin to mitochondria appears to be necessary for the opening of the mitochondrial permeability transition pore and subsequent release of cytochrome c. Only dephosphorylated cofilin translocated to mitochondria resulting in cytochrome c release and apoptosis [13]. Consistent with these reports, the dephosphorylation and mitochondrial translocation of cofilin also appears to be necessary for cerulenin-mediated cytochrome c release and apoptosis based on the following findings. First, after cerulenin-induced apoptosis, cofilin was translocated to mitochondria before the release of cytochrome c. Second, cerulenin treatment decreased the levels of phosphorylated cofilin. Third, only dephosphorylated cofilin translocated to mitochondria. Constitutively active cofilin S3A (dephosphorylated) enhanced, whereas the dominant-negative cofilin S3E (phosphorylated) blocked cytochrome c release and apoptosis. To the best of our knowledge, this is the first report to demonstrate that cerulenin-mediated dephosphorylation of cofilin at Ser 3 is required for the translocation of cofilin to mitochondria, leading to cytochrome c release and apoptosis.

Our results provide detailed information on the molecular mechanisms by which cerulenin induces apoptosis in human leukemia cells (i.e. by activation of ROCK1, inactivation of Akt, and activation of JNK). In a recent study, cerulenin induced apoptosis in various cancer cell lines through diverse cell signaling pathways including PI3K/Akt, JNK, Hexokinase II, and p53 [6, 7, 23, 24]. However, the functional role of the ROCK1/Akt/JNK signaling pathway in the regulation of dephosphorylation and mitochondrial translocation of cofilin, which results in cerulenin-mediated mitochondrial injury and apoptosis in human leukemia cells, is not yet clear. Our results demonstrate that disruption of the ROCK1/Akt/JNK signaling contributes to the cerulenin-mediated dephosphorylation and mitochondrial translocation of cofilin, cytochrome c release, and apoptosis.

Akt is a serine-threonine kinase that is intimately involved in the regulation of cell survival [25]. It is activated by recruitment to the cell membrane through the actions of PI3K, which in turn is negatively regulated by the PTEN phosphatase [26]. Treatment of cells with cerulenin diminished rather than increased Akt phosphorylation, suggesting that this phenomenon could be attributed to PTEN activation. However, the fact that Jurkat cells do not express wild-type PTEN argues against this notion [27]. A more likely possibility is that cerulenin blocks the actions of PI3K, because Akt is a major downstream target of PI3K [28]. The finding that LY29004, an inhibitor of PI3K, enhanced the lethality of cerulenin through inactivation of Akt and one or more of its downstream targets (e.g., cofilin) is consistent with this concept. In fact, caspase-dependent downregulation of Akt is a well-described phenomenon [29]. The present results indicate that cerulenin induces apoptosis by activating caspase-3 and -9, raising the possibility that Akt inactivation might reflect secondary events that stem from caspase activation. Cotreatment of Jurkat cells with the pan-caspase inhibitor z-VAD-fmk, which abrogated cerulenin-induced cleavage/activation of caspases-3 and -9 and apoptosis, failed to prevent Akt inactivation. This data strongly suggests that factors other than caspase-dependent events are involved in this phenomenon.

Several lines of evidence suggest that inactivation of Akt plays a critical functional role in cerulenin-induced mitochondrial injury and apoptosis in human leukemia cells. LY294002, an inhibitor of PI3K, significantly enhanced the lethality of cerulenin by blocking activation of Akt, whereas constitutive activation of Akt largely reversed the lethal consequences of cerulenin treatment (including cytochrome c release, caspase activation, and apoptosis). Interestingly, co-administration of the PI3K inhibitor LY294002, which potentiates inactivation of Akt, enhanced cerulenin-induced JNK activation. Furthermore, constitutive activation of Akt prevented the striking increase in JNK activation in response to cerulenin treatment, raising the possibility that one of the mechanisms by which Akt protects cells from cerulenin lethality is by opposing JNK activation. This phenomenon might be explained by the following data. First, ASK-1, which activates JNK, is a target of Akt inhibitory phosphorylation. Specifically, increased Akt activity might lead to suppression of the JNK activity mediated by ASK1, which could provide a direct link between Akt and JNK [25]. Second, the interaction between Akt and JIP1 inhibited JIP1-mediated potentiation of JNK activity by decreasing JIP1 binding to specific JNK pathway kinases, suggesting that the interaction between Akt and JIP1 acts as a negative switch for JNK activation [30].

Our results demonstrate that activation of ROCK1 has a critical role in the regulation of Akt inactivation, JNK activation, cofilin dephosphorylation and mitochondrial translocation, and mitochondrial apoptosis. Recent studies have shown that ROCK1 regulates apoptosis in various cell types and animal disease models [17–19]. In fact, the caspase-dependent cleavage/activation of ROCK1 is a well-described phenomenon [17]. However, a pan-caspase inhibitor z-VAD-fmk failed to prevent cerulenin-mediated ROCK1 activation, arguing that factors other than caspase-dependent events are involved. Several studies have demonstrated that Akt is a novel ROCK1 downstream effector that regulates cell proliferation and apoptosis [31]. PTEN was recently identified as a ROCK1 substrate that is also involved in cell death and survival [32]. PTEN is a negative regulator of the PI3K/Akt pathway, and PTEN phosphorylation by ROCK1 stimulates PTEN phosphatase activity [33]. However, the fact that Jurkat cells do not express wild-type PTEN argues against this notion [27]. It is likely that inactivation of the Akt/JNK cascade acts downstream of the ROCK1 pathway based on the following evidence. First, pretreatment of cells with the ROCK1 inhibitor Y27632 dramatically abrogated cerulenin-mediated Akt inactivation and JNK activation. Second, knockdown of ROCK1 by siRNA blocked cerulenin-mediated Akt inactivation and JNK activation. Finally, inhibition of ROCK1 with Y27632 or knockdown with siRNA attenuated the cerulenin-mediated dephosphorylation and mitochondrial translocation of cofilin, cytochrome c release, and apoptosis.

In summary, our results indicate that cerulenin induces mitochondrial injury and apoptosis in transformed and primary human leukemia cells *in vitro*, and inhibits tumor growth in a Jurkat xenograft mouse model *in vivo*. Collectively, these findings suggest a hierarchy of events in cerulenin-induced apoptosis in which ROCK1 activation is the primary insult that leads to Akt inactivation and JNK activation, dephosphorylation of cofilin and mitochondrial translocation, mitochondrial injury, cytochrome c release, and apoptosis. These findings could provide a novel mechanistic basis for the application of cerulenin in the treatment of hematologic malignancies.

## MATERIALS AND METHODS

### Chemicals and reagents

Cerulenin was purchased from Sigma (St Louis, MO, USA). LY294002, SP600125, and z-VAD-fmk were purchased from EMD Biosciences (La Jolla, CA, USA). Antibodies against Akt, phosphorylated JNK, JNK1, and β-actin were purchased from Santa Cruz Biotechnology (Santa Cruz, CA, USA). Antibodies against cleaved-caspase-3, cleaved-capase-9, phosphorylated Akt were purchased from Cell Signaling (Beverly, MA, USA). The antibody against FAS was purchased from BD Biosciences PharMingen (San Diego, CA, USA). The antibody against PARP was purchased from Biomol (Plymouth Meeting, PA, USA). Protein concentrations were determined using the BCA (Pierce, Rockford, IL, USA).

### Cell culture and transfection

Human acute myeloid leukemia U937 cells, T-lymphoblastic leukemia Jurkat cells, and acute promyelocytic leukemia HL-60 cells were obtained from American Type Culture Collection (ATCC, Manassas, VA, USA) and cultured in RPMI 1640 medium supplemented sodium pyruvate, sodium bicarbonate, non-essential amino acids, L-glutamine, penicillin, streptomycin, and 10% fetal bovine serum (FBS). The constitutive active form of Akt and the dominant negative Akt mutant (Akt-DN) were kindly provided by Dr. Richard Roth (Stanford University School of Medicine, Stanford, CA, USA), and were sub-cloned into the pcDNA3.1 vector. Jurkat cells were stably transfected with the constitutively active form of Akt and Akt-DN using the Amaxa nucleofector^™^ (Koeln, Germany) according to the manufacturer's recommendations. Stable single cell clones were selected in the presence of 400 μg/mL of geneticin. The expression of Akt in each cell clone was analyzed by western blot analysis as described below.

Peripheral blood samples for *in vitro* studies were obtained from 15 patients with newly diagnosed or recurrent AML after informed consent. Approval was obtained from the Southwest Hospital (Chongqing, China) Institutional Review Board for all studies. AML blasts were isolated using density gradient centrifugation over Histopaque-1077 (Sigma Diagnostics, St Louis, MO, USA) at 400 × g for 38 minutes. Isolated mononuclear cells were washed and assayed for total cell number and viability using trypan blue exclusion. Blasts were suspended at 8 × 10^5^/mL and incubated in RPMI 1640 medium containing 10% FBS in 24-well plates. CD34^+^ cells from bone marrow mononuclear cells of healthy donors were isolated using the MACS cell isolation kit (Miltenyi Biotec, BG, Germany) according to the manufacturer's instructions. After mononuclear cells were washed and counted, they were suspended at a density of 8 × 10^5^/mL prior to treatment.

### Lentiviral gene transfer and gene silencing

The human JNK1 shRNA (5′-CAGAGAGC TAGTTCTTATGAA-3′) and ROCK1 shRNA (5′- TAAAT CGGGTACAACTGGTGC-3′) were synthesized and subcloned into the pLKO.1 plasmid. Plasmids were co-transfected with lentiviral packaging vectors (pLP1, pLP2, and pLP/VSVG) into 293FT cells using Lipofectamine 3000 (invitrogen, Carlsbad, CA, USA) according to the manufacturer's instructions. The lentivirus-containing supernatant was harvested and used to infect Jurkat cells. The cells were subsequently grown under 5 μg/ml puromycin selection to establish stable cell lines.

### Site-directed mutagenesis and transfection

Dephosphorylated (active, S3A) and pseudo phosphorylated (inactive, S3E) human cofilin constructs were a gift from Professor James Bamburg (Colorado State University, Ft. Collins, CO, USA). Plasmids were transfected into Jurkat cells using the Amaxa nucleofector (Koeln, Germany) using the manufacturer's protocols. After 48 h of transfection, the cells were exposed 40 μM cerulenin for 12 h and subsequently subjected to immunoblotting or flow cytometry analysis.

### Preparation of mitochondrial and cytosolic fractions

Mitochondrial and cytosolic fractions were obtained as previously described [31]. Briefly, cell pellets were washed twice with phosphate-buffered saline (PBS) and resuspended in 5× Buffer A (20 mM HEPES, 10 mM KCl, 1.5 mM MgCl_2_, 1 mM EDTA, 1 mM EGTA, 1 mM Na_3_VO_4_, 2 mM leupeptin, 1 mM PMSF, 1 mM DTT, 2 mM pepstatin, and 250 mM sucrose). Cells were homogenized by passing them through a 22-gauge needle 25 times. The homogenate was centrifuged in three sequential steps: 1000 × g, 10,000 × g, and 100,000 × g. The 10,000 × g pellet was considered the mitochondrial fraction, and the 100,000 × g supernatant the cytosolic fraction. Both fractions were subjected to western blot analyses.

### Detection of apoptosis by flow cytometry

Cells were stained with annexin V-FITC and PI to evaluate apoptosis by flow cytometry according to the manufacturer's protocol (BD Biosciences PharMingen). In brief, 1 × 10^6^ cells were washed twice with ice-cold PBS and stained with 5 μL of annexin V-FITC and 10 μL of 5 μg/mL PI in 1× Binding buffer (10 mM HEPES, pH 7.4, 140 mM NaOH, and 2.5 mM CaCl_2_) for 15 min at room temperature in the dark. Quantification of apoptotic cells was performed by flow cytometry using a FACScan cytofluorometer (BD Biosciences). Annexin V-positive cells that did not take up PI were identified as early apoptotic, whereas annexin V-positive cells that were also stained with PI were classified as late apoptotic. The combined percentage of both cell populations was used to calculate the extent of apoptosis after subtraction of the corresponding background values.

### Western blot analysis

Western blot analysis was performed using the NuPAGE Bis-Tris electrophoresis system (Invitrogen, Carlsbad, CA, USA). Total cellular samples were washed twice with ice-cold PBS and lysed in 1× NuPAGE LDS sample buffer supplemented with 50 mM DTT (Fisher Scientific Co., Pittsburgh, PA). The protein concentration was determined using Coomassie Plus Protein Assay Reagent (Pierce). Total cellular protein extracts were separated by SDS-PAGE and transferred to PVDF membranes in 20 mM Tris-HCl, pH 8.0 containing 150 mM glycine and 20% (v/v) methanol. Membranes were blocked with 5% fat-free dry milk in 1× Tris-buffered saline (TBS) containing 0.05% Tween 20 and incubated with the appropriate antibodies. Protein bands were detected by incubating the membranes in HRP-conjugated secondary antibodies (Kirkegaard and Perry Laboratories, Gaithersburg, MD), visualized by Western Lightning^™^ Chemiluminescence Reagent Plus (Perkin Elmer Life Science, Boston, MA, USA), and exposed to X-ray film. All experiments on Western blot analysis were performed three times.

### Xenograft assay

Nude mice mice (5 weeks old) were purchased from Vital River Laboratories (VRL, Beijing, China). All animal studies were conducted according to protocols approved by the Institutional Animal Care and Use Committee of the University. Jurkat cells (2 × 10^6^/0.2 mL/mouse) were suspended in sterile PBS and injected subcutaneously into the right flank of each mouse. Mice were randomized into two groups (15 mice/group). Three days after tumor inoculation, the treatment group received cerulenin (50 mg/kg, intraperitoneally for 6 weeks). The control group received an equal volume of solvent. Tumor size and body weight were measured after treatment at various time-points throughout the study. At the end of the experiment, the mice were sacrificed, 24 h after the last administration of the compound. The tumors were excised, weighed, and fixed in paraformaldehyde. Paraffin-embedded tissues were sectioned and processed for H & E staining, TUNEL, and immunohistochemical staining.

### TUNEL assays

Apoptotic cells were detected in tissue samples using an *In Situ* Cell Death Detection kit (Roche, Mannheim, Germany) according to the manufacturer's protocols. After deparaffinization and permeabilization, the tissue sections were incubated in proteinase K for 15 min at room temperature. The sections were then incubated with the TUNEL reaction mixture, which contains terminal deoxynucleotidyl transferase (TdT) and fluorescein-dUTP, at 37°C for 1 h. After washing three times with PBS, the sections were incubated with the Converter-POD which contained an anti-fluorescein antibody conjugated to HRP (POD) at room temperature for 30 min. After washing three times with PBS, the sections were incubated with 0.05% 3-3′-diaminobenzidine tetrahydrochloride (DAB) and analyzed under a light microscope.

### Histological and immunohistochemical evaluation

Upon completion of the experiments, tumor tissues from representative mice were sectioned, embedded in paraffin, and stained with H & E for histopathologic evaluation. For immunohistochemical analysis, tissue sections (4 μm thickness) were dewaxed and rehydrated in xylene and graded alcohols. Antigen retrieval was performed with 0.01 M citrate buffer at pH 6.0 for 20 min in a 95°C water bath. Slides were allowed to cool for another 20 min, and then rinsed sequentially in PBS and TBS-Tween buffer. Endogenous peroxidase activity was quenched by incubation in TBS-T containing 3% hydrogen peroxide. Each incubation step was performed at room temperature and was followed by three sequential washes (5 min each) in TBS-T. After blocking with 10% goat serum for 1 h, sections were incubated with primary antibodies overnight, washed three times in PBS, incubated with biotinylated secondary antibodies for 1 h, and then incubated with a streptavidin-peroxidase complex for an additional 1 h. After three additional washes in PBS, DAB working solution was applied. Finally, the slides were counterstained in hematoxylin.

### Statistical analyses

In the analyses of apoptosis, the values were presented as the mean ± standard deviation (s.d.). Statistical differences between the control and treatment groups were evaluated using Student's *t*-tests. Differences were considered statistically significant for values *P* < 0.05 or *P* < 0.01.
